# Accelerated lymph flow from infusion of crystalloid fluid during general anesthesia

**DOI:** 10.1186/s12871-024-02494-w

**Published:** 2024-03-27

**Authors:** Robert G. Hahn

**Affiliations:** Department of Clinical Sciences, Karolinska Institutet at Danderyd Hospital, Stockholm, 182 88 Sweden

**Keywords:** Blood, Hemodilution, Fluid balance, Infusion, Pharmacokinetics, Ringer’s solution, Plasma albumin, General anesthesia

## Abstract

**Background:**

Kinetic analysis of crystalloid fluid yields a central distribution volume (*V*_c_) of the same size as the expected plasma volume (approximately 3 L) except during general anesthesia during which *V*_c_ might be only half as large. The present study examined whether this difference is due to influence of the intravascular albumin balance.

**Methods:**

A population volume kinetic analysis according to a three-compartment model was performed based on retrospective data from 160 infusion experiments during which 1–2.5 L of crystalloid fluid had been infused intravenously over 20–30 min. The plasma dilution based on blood hemoglobin (Hb) and plasma albumin (Alb) was measured on 2,408 occasions and the urine output on 454 occasions. One-third of the infusions were performed on anesthetized patients while two-thirds were given to awake healthy volunteers.

**Results:**

The Hb-Alb dilution difference was four times greater during general anesthesia than in the awake state (+ 0.024 ± 0.060 versus − 0.008 ± 0.050; mean ± SD; *P* < 0.001) which shows that more albumin entered the plasma than was lost by capillary leakage. The Hb-Alb dilution difference correlated strongly and positively with the kinetic parameters governing the rate of fluid transfer through the fast-exchange interstitial fluid compartment (*k*_12_ and *k*_21_) and inversely with the size of *V*_c_. Simulations suggest that approximately 200 mL of fluid might be translocated from the interstitial space to the plasma despite ongoing fluid administration.

**Conclusions:**

Pronounced plasma volume expansion early during general anesthesia is associated with a positive intravascular albumin balance that is due to accelerated lymphatic flow. This phenomenon probably represents adjustment of the body fluid volumes to anesthesia-induced vasodilatation.

**Supplementary Information:**

The online version contains supplementary material available at 10.1186/s12871-024-02494-w.

## Background

Volume kinetics is a macroscopic method that has been used in anesthesia research over the past 25 years for analyzing the distribution of infusion fluids [1]. Certain features differ between the kinetic output from experiments performed in the awake and anesthetized state. The most notable difference is that the diuretic response to infused fluid is much lower during general anesthesia [1]. Another discrepancy is the size of the central volume (*V*_c_), which is close to the expected plasma volume when crystalloid fluid is infused in awake volunteers [2] but is smaller during general anesthesia [3]. The lowest values are found during laparoscopy in which *V*_c_ might be only half as large as found in awake volunteers [3, 4]. This deviation is counterintuitive as anesthesia-induced vasodilatation is expected to produce an enlarged *V*_c_. An explanation for this contradictory finding would interest anesthesia-related fluid physiology and therapy researchers.

The *V*_c_ parameter is not a true measure of the plasma volume but is rather a conversion factor between plasma dilution and modeled plasma volume expansion. A small *V*_c_ indicates that the measured plasma dilution is greater than the expected dilution based on the infused fluid volume. A deviation of this kind occurs if fluid is attracted from an extravascular source, such as by accelerated lymphatic flow or recruitment of fluid from the intravascular space. The plasma dilution may then become unexpectedly large [5, 6].

The aim of the present study was to explore whether the intravascular albumin balance explains the low *V*_c_ found during fluid therapy in anesthetized patients. The study used albumin as biomarker of lymphatic flow since the lymph is rich in albumin. For this purpose, volume kinetic analyses of crystalloid fluid experiments in which blood hemoglobin (B-Hb) and plasma albumin (P-Alb) measured concurrently has been applied. The plasma dilution based on B-Hb minus the plasma dilution based on P-Alb would be zero if the inflow and outflow of albumin to the plasma were perfectly matched. In contrast, the difference would be positive if more albumin entered the plasma than was lost via transcapillary leakage. The hypothesis was that *V*_c_ is influenced by the intravascular albumin balance which, in turn, provides information about the physiological adaptation to general anesthesia.

## Methods

The study material was derived from a database of intravenous infusion experiments in humans in which the purpose was to study fluid volume kinetics. The studies were planned and supervised by the author using similar standardized protocols and sample collection. The exclusion criteria were age < 18 years and severe cardiac lung, hepatic, or renal disease.

### Included studies

Data were chosen from 160 experiments that were derived from six published studies that included frequent concurrent measurements of both B-Hb and P-Alb during and after intravenous infusion of 15 to 25 mL/kg of various crystalloid fluids over 20–30 min. The cohorts included administration of Ringer’s acetate to healthy volunteers [7, 8], Ringer’s acetate/lactate to patients undergoing surgery (thyroid resection and open hysterectomy) [9, 10], or urological irrigating fluid (glycine, mannitol, and sorbitol) to volunteers [11, 12]. Infused volumes varied between 1.0 and 2.5 L. Sampling continued for 120–240 min after infusion initiation. No vasoactive drugs were given, and no paired measurements were excluded. The B-Hb data have previously been used for kinetic calculations.

### Ethics

All protocols were approved by the appropriate Ethics Committees, and the research was conducted in accordance with the Declaration of Helsinki. The Ethics Committee of Stockholm approved applications with the following identification numbers: 228/98 [7], 54/95 [8], 269/02 [9], 127/92 [11, 12], and the Ethics Committee of Riga Stradins University, Latvia, on 2016-01-27 (no number) [10]. Each subject gave written informed consent to participate before any experiment was initiated.

### Procedures

The volunteers were allowed to ingest one glass of liquid and eat one sandwich to prevent dehydration and hunger stress before coming to the hospital. Patients undergoing general anesthesia were in the fasting state but received premedication with 5 mg diazepam by mouth. All experiments were initiated between 8 and 9 a.m. in a hospital facility, and the subjects rested for 30 min before an infusion was given to reach a steady state. The method for managing anesthesia is detailed in the respective studies [9, 10].

Infusions were administered at a constant rate via an infusion pump. Venous blood for measurement of the B-Hb and P-Alb concentrations was withdrawn every 5 min during the infusion, for 30 min thereafter, and at 15–30 min intervals for a total length of up to 2.5–6 h. The samples were analyzed at the hospital’s clinical chemistry laboratory with coefficients of variation of approximately 1% and 2%, respectively. The volunteers voided in a plastic bucket whenever needed while remaining supine. The urine excreted by anesthetized patients was collected via an indwelling bladder catheter. All collected urine was measured to the nearest 25 mL. Arterial pressures were measured non-invasively with an automatic device. The mean arterial pressure (MAP) was reported.

### Kinetic model

A volume kinetic model with three functional fluid compartments, five fixed rate constants (*k*_12_, *k*_21_, *k*_23_, *k*_32_, and *k*_10_), and one scaling factor between dilution and volume (*V*_c_, central volume) was fitted to the dependent variables, which were the frequently measured plasma dilution and the urinary excretion [13, 14].

**Fig. 1:**
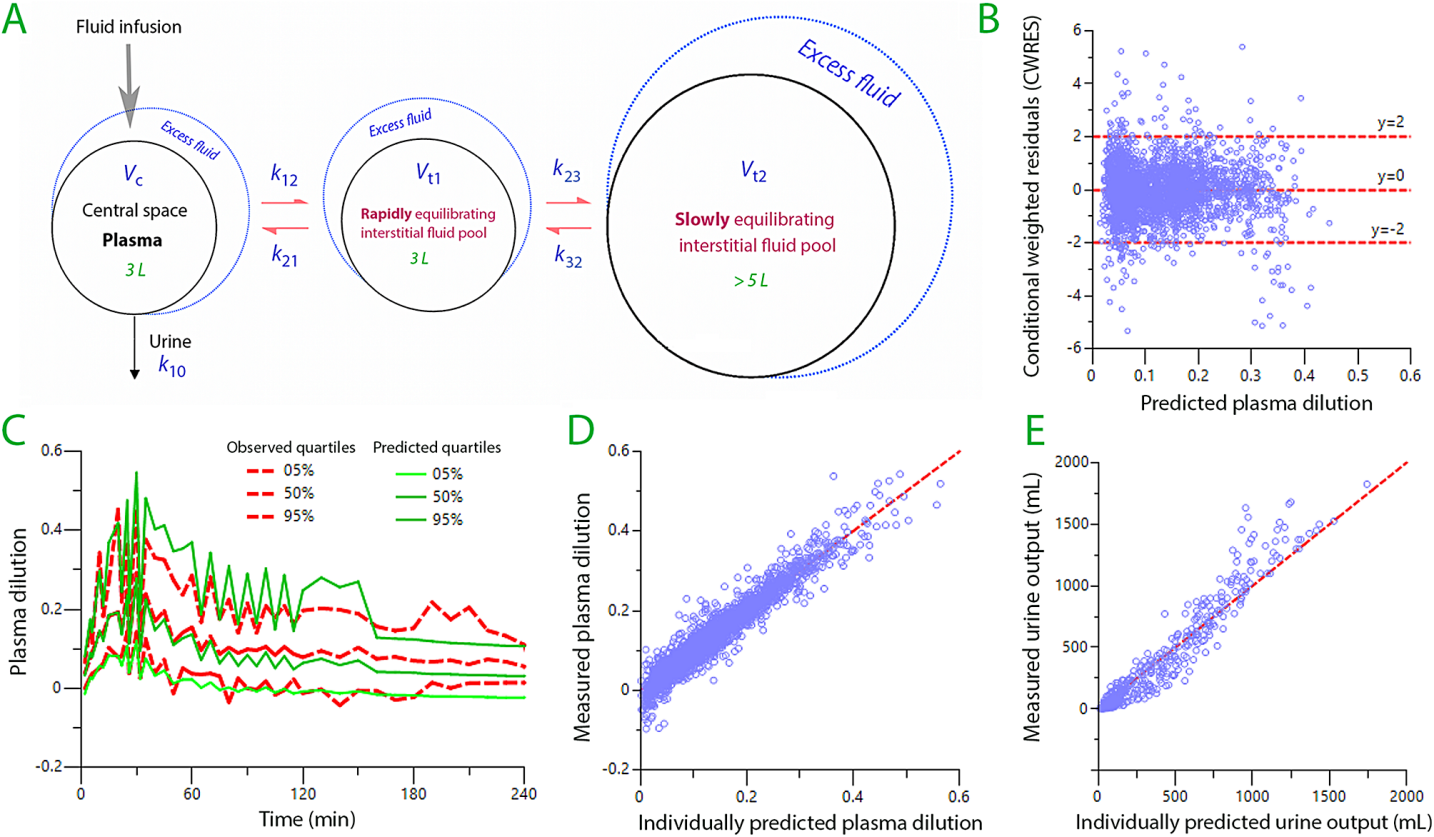
**(A)** Schematic drawing of the used kinetic model. **(B)** Conditional weighted residuals (CWRES) *versus* the plasma dilution as predicted by the kinetic model without covariates. Good model specification is indicated by random distribution of the data around the individual predictions and over time, and by the few data points > ± 3 standard deviations. **(C)** Predictive check. The confidence limits for the plasma dilution as given by the observed data and when these data were recreated by 1,000 simulations using the kinetic parameters. The hatched pattern is due to irregular sampling frequencies between studies. **(D)** The measured plasma dilution *versus* the model-predicted plasma dilution when all covariates were considered (*N* = 2,408). The line of unity is shown in red. **(E)** Same plot for the urinary excretion (*N* = 454)

A schematic drawing is shown in Fig. 1A. Fluid was infused at a rate *R*_o_ into the plasma (*V*_c_) from which distribution occurred via a rate constant (*k*_12_) to a fast-exchange interstitial space (*V*_t1_) and further to a slow-exchange compartment (*V*_t2_) at a rate governed by a rate constant, *k*_23_. Return flow from *V*_t2_ to *V*_t1_ occurred at a rate given by *k*_32_ and flow from *V*_t1_ to *V*_c_ by a rate constant *k*_21_.

The plasma dilution based on B-Hb, which is the hemodilution divided by (1 – baseline hematocrit), indicated the volume expansion of *V*_c_ resulting from the infusion. Division by the hematocrit was applied to interpret Hb changes during the experiment in terms of the relationship between red blood cell mass and plasma volume. The plasma dilution was derived by calculating the dilution of the P-Alb concentration without correction for the hematocrit.

The elimination rate constant, *k*_10_, was taken as the measured urine output divided by area under the curve for the volume expansion of *V*_c_. The differential equations for the kinetic model are shown in Supplementary File 1.docx.

### Covariate analysis

The six fixed parameters in the kinetic “base model” (*k*_12_, *k*_21_, *k*_23_, *k*_32_, *k*_10_, and *V*_c_) were concurrently fit to all measurements of plasma dilution and urinary excretion (dependent variables) using the Phoenix software version 8.3.4 for nonlinear mixed effects (Phoenix NLME, Pharsight, St. Louis, MO) with the First Order Conditional Estimation Extended Least Squares (FOCE ELS) as search routine. The analysis is based on likelihood mathematics and identifies the typical value of each model parameter (“theta:s”) and two levels of random effects (“eta:s” = differences between individuals/occasions, and epsilon = the residual variability). In the present study, the typical value of each model parameter (“theta”) is reported together with its 95% confidence interval (CI), while the “eta:s” were used to search for variables that could serve as individual-specific *covariates*.

The precision of the six parameters in the “base model” to predict the input data could potentially be improved by the addition of such covariates. The most promising candidates were tested, one by one, by adding them to the base model in a diagonal design, which assumes no correlation between random effects. Ten variables were considered for inclusion: (1) age, (2) male/female, (3) body weight, (4) post-infusion/ongoing infusion, (5) Ringer’s/irrigating fluid, (6) acetate/lactate as buffer, (7) awake/general anesthesia, (8) blood loss, (9) MAP, and (10) the Hb-Alb dilution difference. The last two were used as “time-varying covariates”, which means a new value was entered for each time point. How a covariate changes the fixed parameters is explained in Supplementary File 1.docx.

### Statistics

Demographic data were reported as the mean ± standard deviation (SD). Correlations between variables were studied by simple linear regression analysis and comparisons of data between study groups by one-way analysis of variance. Kinetic parameters were given as the best estimate and 95% CI. The criterion for including a covariate was that its inclusion should reduce the − 2 log likelihood for the kinetic model by > 6.6 points (*P* < 0.01) and that the 95% CI for the estimate could not include 0. A chi-square *P*-value was given by the Phoenix program based on the Likelihood Ratio Test.

The behavior of the kinetic base model (goodness-of-fit) was studied by plotting the relationship between the weighted conditional residuals and the predicted plasma dilution. The performance of the model with covariates was studied by comparing the measured and simulated values over time (“predictive check”) and by comparing gradually higher measured and plasma dilution and urine output values regardless of time (residual plots).

## Results

### Demographic*s*

The analysis comprised 2,408 matched pairs of B-Hb and P-Alb (mean, 15 per experiment) and 454 urine collections (2.8 per experiment). The participants had a mean ± SD age of 43 ± 16 years and body weight of 76 ± 11 kg. They received 1,575 ± 419 mL of fluid at a rate of 60 ± 9 mL/min. MAP was measured on 1,548 occasions and averaged 81.6 ± 16.3 mmHg. 31% of the participants were females.

### Kinetic analysis

The 6 fixed parameters were successfully estimated by the kinetic model. A schematic drawing of the kinetic model and measures of the model’s performance are shown in Fig. 1.

The *covariate analysis* showed that the Hb-Alb dilution difference was associated with higher *k*_12_ and *k*_21_ and lower *V*_c_, which present evidence for accelerated passage through the fast-exchange compartment, *V*_t1_.

The kinetic model was also corrected for previously known covariate effects; low MAP retarded the urine flow (low *k*_10_) [3], a large body weight caused an increase in *V*_c_ [3], and general anesthesia had a slight own effect on *k*_10_ (in addition to an effect via MAP) [13].

Further analyses confirmed that “ongoing infusion”, as opposed to the post-infusion period, was associated with a limited entrance of fluid into the slow-exchange compartment (low *k*_23_) and a moderate decrease of the return flow to the plasma (low *k*_21_) [14].

The final parameter estimates are shown in Table 1 and the original data used for the kinetic analysis given in Supplementary file 2.xls.

**Table 1:** Kinetic parameter values. Population kinetic parameters for infused Ringer’s solution in 160 conscious and anesthetized subjects. Typical values (tv) for the fixed parameters for all subjects, followed by individual-specific covariates are shown

Kinetic parameter	Covariate	Best estimate	95% CI	CV%	*P*-value
***Fixed***					
tv*V*_c_ (L)		3.21	2.90–3.54	5.4	
tv*k*_12_ (10^− 3^ min^− 1^)		113.0	77.1–148.0	16.1	
tv*k*_21_ (10^− 3^ min^− 1^)		118.8	58.3–179.4	26.0	
tv*k*_23_ (10^− 3^ min^− 1^)		24.0	20.1–28.0	8.4	
tv*k*_32_ (10^− 3^ min^− 1^)		64.0	37.8–90.3	20.9	
tv*k*_10_ (10^− 3^ min^− 1^)		20.7	17.5–24.1	8.1	
***Covariates***					
*k*_10_	MAP (mmHg)	2.10	1.52–2.68	14.1	1.6 × 10^− 13^
*V*_c_	Body weight (kg)	0.75	0.56–0.95	13.4	3.5 × 10^− 8^
*k*_10_	General anesthesia (no/yes)	-1.67	-1.98 to -1.37	-9.4	< 1 × 10^− 3^
*k*_21_	Hb-Alb dilution difference	3.08	2.00–4.15	17.8	5.9 × 10^− 5^
*k*_12_	Hb-Alb dilution difference	2.86	1.85–3.69	18.0	1.1 × 10^− 12^
*V*_c_	Hb-Alb dilution difference	-1.06	-1.56 to -0.55	-24.4	1.3 × 10^− 53^
*k*_21_	Ongoing infusion (no/yes)	-0.50	-0.70 to -0.30	-20.7	3.0 × 10^− 19^
*k*_23_	Irrigating fluid (no/yes)	1.22	0.85–1.60	15.6	2.4 × 10^− 5^
*k*_23_	Ongoing infusion (no/yes)	-7.60	-8.49 to -6.70	-6.0	2.6 × 10^− 12^
*V*_c_	Sex (male/female)	-0.50	-0.62 to -0.38	-12.1	< 1 × 10^− 3^
*k*_21_	General anesthesia (no/yes)	-0.44	-0.61 to -0.29	-18.2	< 1 × 10^− 3^

CI = confidence interval. CV% = coefficient of variation (inter-individual)

*P*-values denote strengthening of the model for each added covariate. Covariate models: Power = MAP and body weight; Linear = Hb-Alb dilution difference; Exponential = the others.

### Simulations

**Fig. 2:**
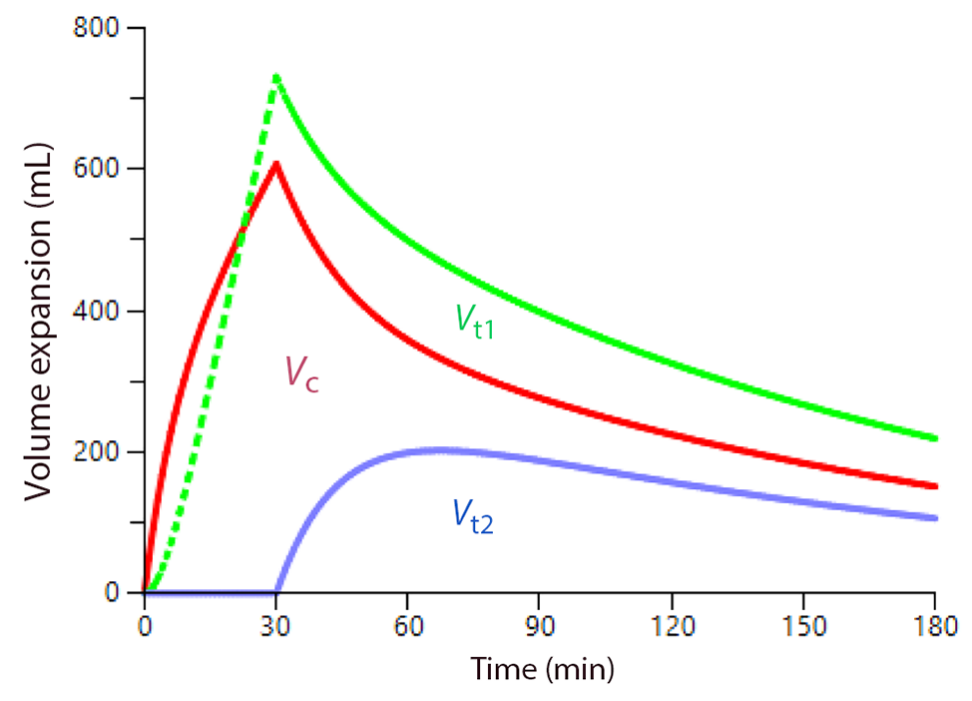
Distribution of Ringer solution. Simulation of the distribution of an infusion of 1,575 mL of Ringer’s solution at a constant rate over 30 min. *V*_c_ = red line, the plasma compartment. *V*_t1_ = green line, the fast-exchange interstitial compartment, and *V*_t2_ = blue line, the slow-exchange interstitial compartment. The urine output is not shown. The graph is based on all 160 experiments. The separation between awake and anesthetized participants necessitated a correction of *k*_10_ for the effect of general anesthesia per se and of different MAPs, which was 92 mmHg for awake volunteers (*N* = 106), 75 mmHg for anesthetized patients (*N* = 54), and an overall mean of 82 mmHg

The implications of the kinetic output are illustrated using simulations from the Phoenix software. Figure 2 shows how the infusion of 1,575 L of Ringer’s over 30 min distributes between the three body fluid spaces when using the data based on all 160 experiments.

**Fig. 3:**
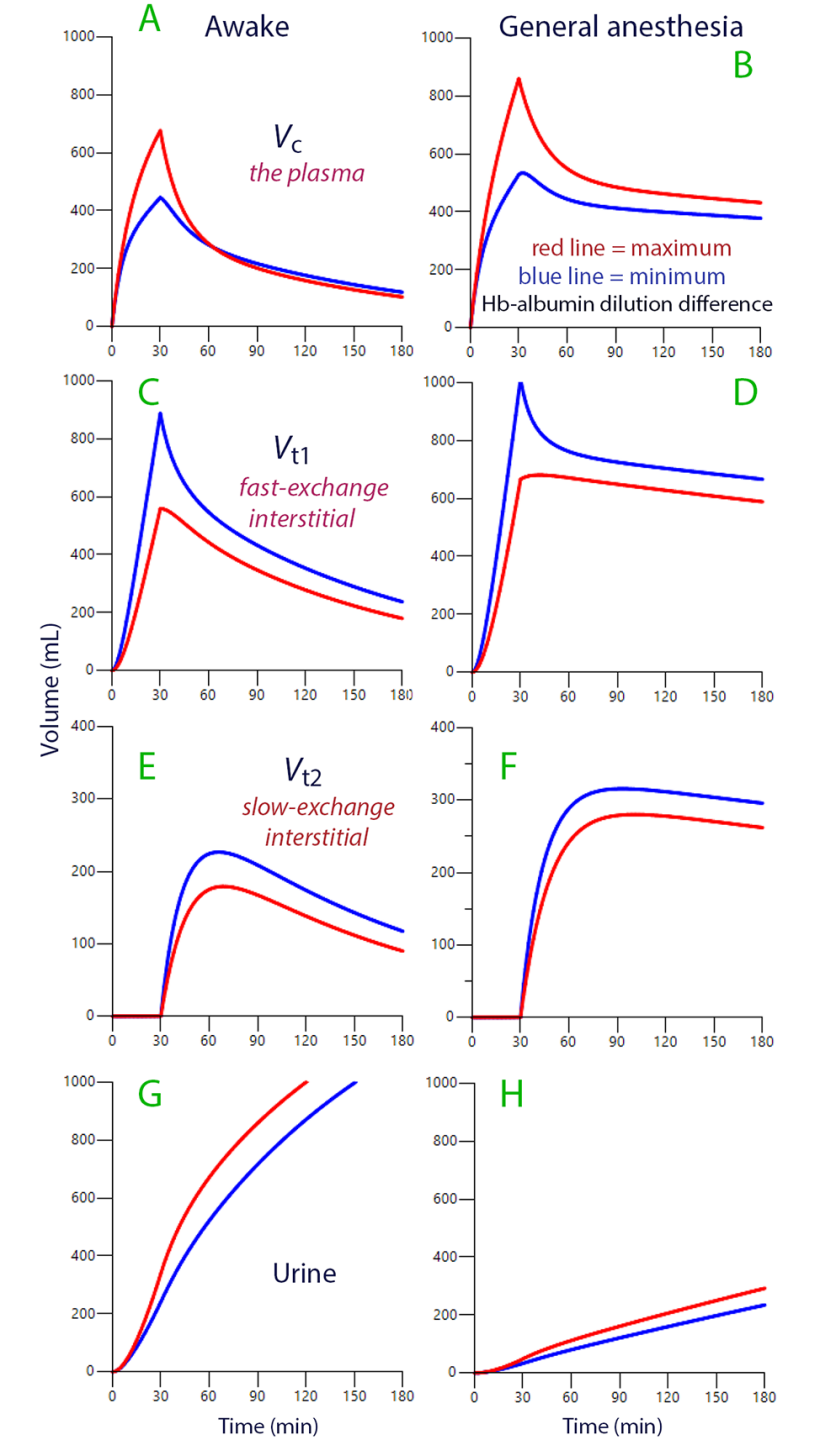
Interstitial washdown. Simulations of the distribution of an infusion of 1,575 mL of Ringer’s solution rate over 30 min divided on awake (left column; **A**, **C**, **E**, and **G**) and anesthetized (right column; **B**, **D**, **F**, and **H**) participants. The red lines were created by the data obtained with a Hb-Alb dilution difference of + 0.2 and the blue line with a difference of − 0.15

The Hb-Alb dilution difference for all 2,408 matched pairs of B-Hb and P-Alb varied between − 0.15 and + 0.20 dilution units (mean value + 0.004, 20 extreme outliers excluded). Figure 3 illustrates the influence of these minimum and maximum Hb-Alb dilution differences on the distribution of infused fluid between the three body compartments.

The flow rates between the body compartments differed greatly depending on the Hb-Alb dilution difference. For example, three times as much fluid had entered *V*_c_ from *V*_t1_ at 60 min in the those with a high (+ 0.2) than with a low (–0.15) Hb-Alb dilution difference (Supplementary File 3.xls). However, these differences in flow were not fully reflected in the expansion of the body fluid compartments (Fig. 3**)**, which was due to compensation by higher values of *k*_12_.

### Albumin-induced distortion of *V*_c_

**Fig. 4:**
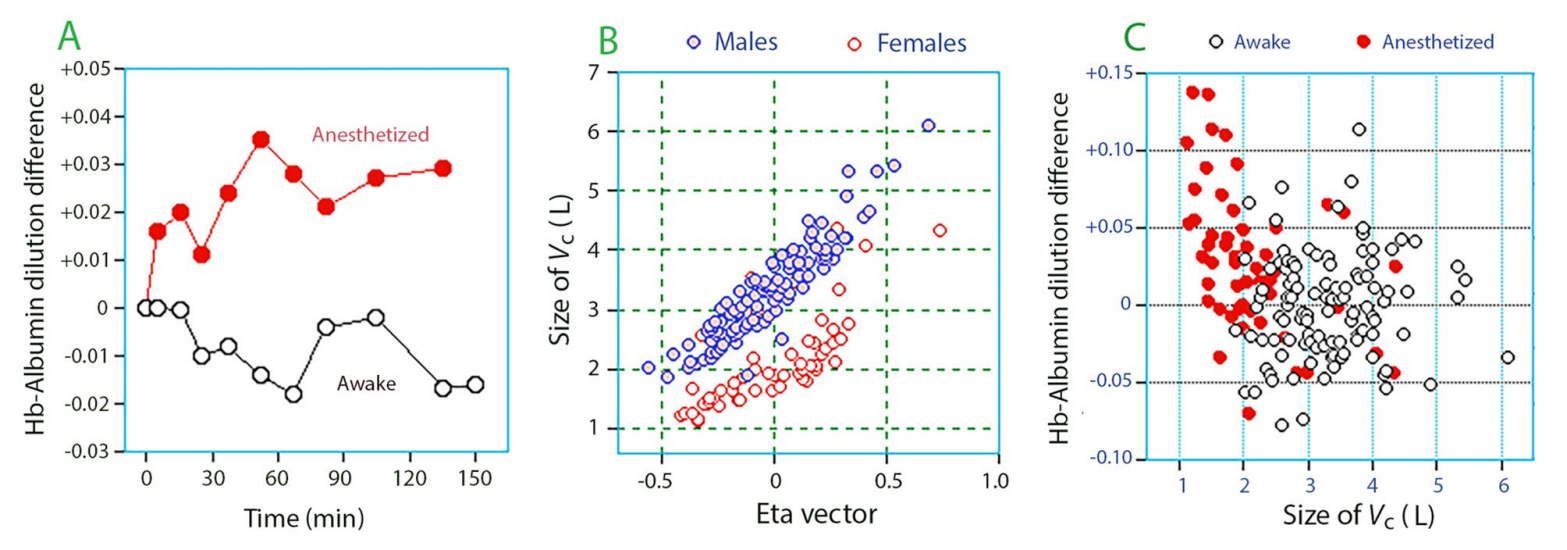
Blood hemoglobin albumin (Hb-Alb) dilution difference. **(A)** The Hb-Alb dilution difference in anesthetized and awake subjects over time. Mean values. The standard deviation was large, approximately 0.05 for all data points (not shown). **(B)** The eta vector (L1 random effects parameter) for the relationship between the Hb-Alb dilution difference and *V*_c_ depending on sex. Each point is one experiment. Eta = 0 was approximately 3 L for males and 2 L for females. **(C)** The size of the central volume (*V*_c_) *versus* the mean Hb-Alb dilution difference for each participant during the first hour of the study

Based on all data points, the mean Hb-Alb dilution difference was four times greater during general anesthesia than in the awake state (+ 0.024 ± 0.060 versus − 0.008 ± 0.050; *P* < 0.001). The distribution of the data is shown in Fig. S1 of Supplementary File 1.docx. Separation of the mean values became apparent soon after the infusions were initiated, although between-subject variability was great (Fig. 4A).

The body weight and the Hb-Alb dilution difference was the strongest combination of factors that explained the between-subject variability of *V*_c_ in males and females (Fig. 4B). No intrinsic effect of general anesthesia had to be considered.

**Fig. 5:**
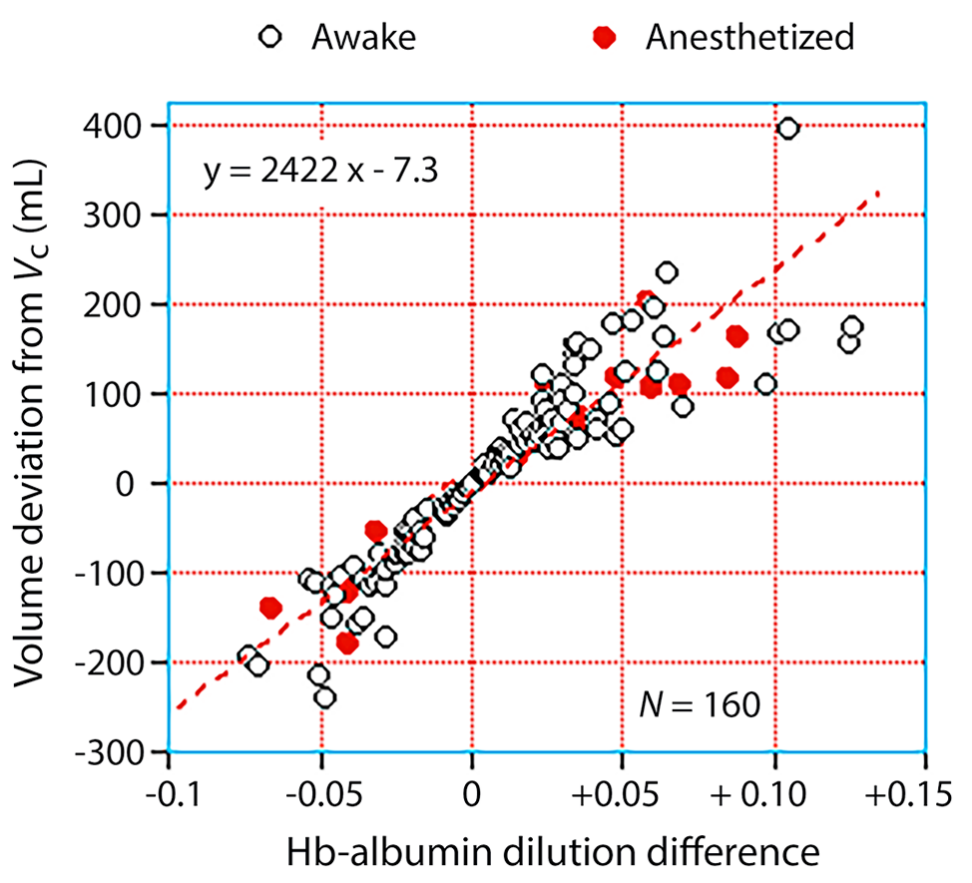
Correction of the central volume. The mean Hb-Alb dilution difference for each infusion experiment *versus* the size of the central volume and the modeled *V*_c_ is compared by linear regression analysis. The latter variable was taken as the product of x and (1+y) in Fig. 4C

The minimum and maximum Hb-Alb dilution difference (–0.15 and + 0.20, respectively) caused a maximum distortion in *V*_c_ by approximately 500 mL in either direction from the overall mean of 3 L (Table 2). However, these extremes were present only during a limited time of an experiment. A more clinically relevant distortion is given by the relationship between the modeled *V*_c_ and the mean Hb-Alb dilution difference during *entire* experiments, which is shown in Fig. 4C. The product of these variables yielded the average deviation of the “true” central volume from the modeled *V*_c_; this deviation varied between approximately − 200 and + 200 mL, and the linear relationship was the same for awake and anesthetized participants (Fig. 5). The average deviation was + 1 ± 101 mL in the awake volunteers and + 38 ± 91 mL in the anesthetized patients (*P* < 0.02).

**Table 2:** Deviation of *V*_c_ depending on the blood hemoglobin-albumin (Hb-Alb) dilution difference. **Line (A)** Ranges of Hb-Alb dilution differences. **Line (B)***V*_c_ as given by the covariance between the Hb-Alb dilution difference and the typical value (tv) of *V*_c_ after considering the difference in body weight between the groups. **Line (C)** The volume that corresponds to the deviation of the assumed albumin balance. **Line (D)** The size of *V*_c_ if the Hb-Alb dilution difference had been zero

	Awake		Anesthesia	
A. Modeled difference	-0.15	+ 0.20	-0.15	+ 0.20
B. *V*_c_ (L)	3.81	2.55	3.58	2.40
C. Volume (= A x B)	-0.57	+ 0.51	-0.54	+ 0.48
D. Corrected *V*_c_. (= B-C)	3.24	2.82	3.04	2.88

Based on all four subgroups, C can be estimated as 3000 * A – 105

## Discussion

### Key result

This study shows that recruitment of albumin from the interstitial space modulates the plasma volume expansion when Ringer’s solution is infused. The degree of modulation depends on the rate of fluid transfer across the interstitial fluid compartment that exchanges fluid fast with the plasma (*V*_t1_). Rapid passage transports more interstitial albumin, which results in a positive Hb-Alb dilution difference and, secondarily, in a low *V*_c_. Such accelerated lymphatic flow causes an increase in the plasma volume **(**Fig. 3A, B**)** and counteracts interstitial edema **(**Fig. 3C-F**).**

### Why *V*_c_ decreases

A small *V*_c_ can either be due to hypovolemia or to fluid entering the circulating plasma that is not considered by the kinetic model. For example, *V*_c_ is markedly reduced when hypertonic saline is infused because it withdraws extravascular fluid to the plasma [15]. The present study focused on nearly isotonic crystalloid fluid and identified accelerated lymphatic flow as the cause of a small *V*_c_. This conclusion is based on the covariance relationships between the Hb-Alb dilution difference and *V*_c_, *k*_12_, and *k*_21_.

The capillary leakage of albumin and lymphatic return do not always agree in the short perspective, but they are usually in good balance when studied over 3–4 h in awake humans [16]. Partial derivatives show that *V*_c_ is primarily estimated during the first hour of a 30-min infusion experiment; therefore, an estimate of *V*_c_ is most affected by the Hb-Alb dilution difference during that time.

In the present study, the mean Hb-Alb dilution difference was only slightly negative in the awake volunteers (–0.008 dilution units) and the mean modeled deviation from the correct *V*_c_ negligible. However, an increase of the Hb-Alb dilution difference occurred more frequently during anesthesia, indicating accelerated passage through the fast-exchange interstitial fluid compartment (*V*_t1_). One reason for the faster passage might be a higher capillary pressure during anesthesia due to a strong inhibition of urine output (cf. Figure 3D and E).

### Fast interstitial passage

The fast-exchange interstitial fluid compartment (*V*_t1_) behaves like a telephone line. The lymphatic flow increases within minutes when transcapillary leakage of fluid is accelerated by volume loading with crystalloid fluid [17]. Molecules still require many hours to pass, which causes some of the interstitial fluid to be replaced by recently filtered fluid. However, the balance between capillary leakage and lymphatic return becomes disturbed more easily for albumin than for fluid. The fluid rates can change quickly while the capillary leakage of albumin is slow and tightly controlled regardless of how fast fluid passes through the interstitium. This situation is the physiological background of the positive Hb-Alb dilution difference during anesthesia.

The time required for a molecule to travel through *V*_t1_ at steady state can be estimated based on the rate parameters reported in the present study. The baseline capillary leakage of fluid averages 7 mL/min [14], which gives a passage time for a molecule to be 3000/7 min = 7 h (as the size of *V*_t1_ can be estimated from *V*_c_*k*_12_ / *k*_21_ = 3 L). In contrast, the same calculation yields a passage time of only 1.5 h when the plasma volume is expanded by 300 mL. A large amount of albumin then follows the lymph to the bloodstream while the interstitial fluid is gradually replaced.

The physiologist Arthur Guyton regarded the described intravascular enrichment with albumin as an edema-preventing consequence that operates when the capillary leakage is increased. He called it “interstitial washdown” [5].

### How strong is the effect?

The effects of lymphatic albumin on the plasma volume can be quantified by the covariates in the kinetic analysis. *V*_c_ becomes larger than the plasma volume when the Hb-Alb dilution difference is negative and would seem to indicate an excess plasma volume. However, this situation is an illusion because the plasma volume is rather on the low side as the capillary leakage of albumin has exceeded the lymphatic return. In contrast, a positive Hb-Alb dilution difference indicates an inflow of albumin, which dilutes the plasma more than the infused fluid does (Table 1). This situation gives the impression that the plasma volume is small, which would be correct if *V*_c_ was small due to blood loss. However, the opposite case is true here because of the rapid transfer of albumin-rich fluid through the interstitial space.

The maximum deviations in plasma volume were approximately ± 500 mL if we consider the highest and lowest Hb-Alb dilution differences at any time point. Naturally, the extreme values prevailed during only a short period. The deviation in plasma volume from the modeled estimate of *V*_c_ during an entire experiment was in the range of ± 200 mL (Fig. 5**)**.

### Albumin enrichment

The literature addressing “interstitial washdown” is limited, and most data originate from studies of isolated tissues [18–20]. The albumin concentration of the interstitial fluid is approximately half as high as in the plasma [21, 22], but can reach 75% in the thoracic duct [23]. The plasma albumin concentration might increase by 0.3–1.0 g/L during fluid infusion experiments, and the enrichment varies over time. Calculations suggest that the albumin concentration of the lymph in humans decreases by 50–75% within 1–2 h after volume loading [6], which is a finding that agrees with animal experiments [17].

The lymphatic system has active pumping mechanisms. Accelerated transcapillary leakage caused by plasma volume expansion has both inotropic and chronotropic positive effects on lymphatic flow [24, 25]. Lymphatic pumping is inhibited by anesthetic drugs [26–28], which was apparent in our case by a 30% reduction of *k*_21_ in anesthetized patients (Table 1). However, this inhibition was not strong enough to prevent albumin enrichment.

### Kinetic model

The results were derived by population volume kinetics, which is based on monitoring of the hemodilution and urinary excretion during infusion experiments. Dynamic events can be studied and simulated, just as in drug pharmacokinetics, and require minimal invasiveness. The comprehensive collection of data used for the present calculations was made possible by maintaining the same strategy for setting up studies and blood sampling during fluid studies performed over several decades.

The whole-body model uses five rate constants to separates delays in fluid transfer between three functional body fluid spaces [13]. The two interstitial compartments communicate with the plasma via a serial connection [14]. This finding is supported by the fact that the slow-exchange compartment does not become filled with fluid until the fast-exchange compartment has expanded. The former compartment might possibly correspond to the free fluid phase and the latter one to the gel phase of the interstitial space [13].

### Limitations

The study data underlying this report were collected from a database consisting of volume kinetic experiments. Matched pairs of B-Hb and P-Alb were analyzed in a high-quality multichannel analyzer, and the urinary excretion was measured in a bucket or urine bag. The kinetic analysis is based on that dilution of B-Hb and P-Alb both indicate the distribution of the infused fluid volume and not the volume of distribution of the biomarkers.

All covariates that possibly affects the kinetics of crystalloid fluid could not be included in a single analysis. Therefore, studies were included with very small variations in infusion volume or infusion time. Hormones were not measured. However, 12% of the volunteers were hypovolemic, but the impact of blood loss on fluid distribution was slight when stronger covariates were added to the kinetic model. Many experiments with volunteers were made with a urological irrigating solution for which partial intracellular distribution was calculated as a covariate to *k*_23_ and not further discussed. Therefore, the present study should not be considered a strict comparison between crystalloid fluid kinetics in the awake and anesthetized states.

## Conclusion

Accelerated transport of infused crystalloid fluid across a fast-exchange interstitial fluid compartment stabilizes the plasma volume and causes a decrease in peripheral edema. These events lead to an increase in the plasma content of albumin and are more common during general anesthesia than in the awake state. They are indicated by a positive Hb-Alb dilution difference during fluid therapy and a small *V*_c_ in volume kinetic analyses. Both these variables can apparently be used to study the balance between capillary leakage of albumin and the addition of albumin-rich lymph to the plasma.

### Electronic supplementary material

Below is the link to the electronic supplementary material.

Supplementary Material 1

Supplementary Material 2

Supplementary Material 3

## Data Availability

All data generated or analyzed during this study are included in this published article and its supplementary information files.

## Declarations

### Author´s information

Robert G. Hahn is professor emeritus of anesthesia and intensive care at Karolinska Institutet in Stockholm, Sweden.
