# Supplementary material for: Accelerated lymph flow from infusion of crystalloid fluid during general anesthesia
Source: BMC Anesthesiol. 2024 Mar 27;24:119. doi: 10.1186/s12871-024-02494-w (PMC10967119; doi:10.1186/s12871-024-02494-w)
Supplement: Supplementary file 1 — Supplementary Material 1 [file 12871_2024_2494_MOESM1_ESM.docx]

Supplementary File 1

Accelerated lymph flow from infusion of crystalloid fluid during general anesthesia

**_____________________________________________________________________________**

**Ethics approvals**

| Males /  females | Infusions | Surgery | Publication | Ethics approval | Committee |
| --- | --- | --- | --- | --- | --- |
|  |  |  |  |  |  |
| 10 / 0 | 10 | No | Anesthesiology 1999, 90, 81-91. | 54/95 | Huddinge Hospital |
| 10 / 0 | 20 | No | Anesthesiology 2002, 96, 1371-80. | 228/98 | Huddinge Hospital |
| 4 / 25 | 29 ^1^ | Thyroid surgery | Anesthesiology 2005, 103, 460-9. | 269/02 | Huddinge Hospital |
| 0 /25 | 25 | Open hysterectomy | BMC Anesthesiology 2020, 20: 95. | 2016-01-27 ^2^ | Riga Stradins, Latvia |
| 10 / 0 | 41 | No | Scand J Urol Nephrol 1999; 33: 35-41 | 127/92 | Huddinge Hospital |
| 10 / 0 | 20 | No | Acta Anaesthesiol Scand 1991: 35: 725-730. | Not available | Huddinge Hospital |

^1^ one patient with major hemorrhage was excluded.

^2^ the date of the approval is given as no numbering was practiced.

**Kinetic model**

Fluid is infused at the rate *R*_o_ into the plasma (*V*_c_) from which distribution occurs at a rate (*k*_12_) to a rapid-exchange interstitial space (*V*_t1_). The distributed fluid returns to *V*_c_ at a rate determined by a constant *k*_21_. Fluid is also distributed from *V*_t1_ to a slow-exchange interstitial space (*V*_t2_) at a rate determined by a constant *k*_23_. The return of this fluid to *V*_t1_ is governed by a rate constant *k*_32_. Urinary excretion (U) occurs in proportion to the volume expansion of *V*_c_ by a rate constant *k*_10_. The differential equations are:

 d*v*_c_ /dt = *R*_o_ – *k*_12_ (*v*_c_ – *V*_c_) + *k*_21_ (*v*_t1_ – *V*_t1_) – *k*_10_ (*v*_c_ – *V*_c_)

d*v*_t1_ /dt = *k*_12_ (*v*_c_ – *V*_c_) – *k*_21_ (*v*_t1_ – *V*_t1_) – *k*_23_ (*v*_t1_ – *V*_t1_) + *k*_32_ (*v*_t2_ – *V*_t2_)

d*v*_t2_ /dt = *k*_23_ (*v*_t1_ – *V*_t1_) – *k*_32_ (*v*_t2_ – *V*_t2_)

dU /dt = *k*_10_ (*v*_c_ – *V*_c_)

Expanded volumes are indicated by lower-case letters (*v*_c_, *v*_t1,_ and *v*_t2_) and baseline volumes and variables by capital letters (*V*_c_, *V*_t1,_ and *V*_t2_).

Fixed parameters to be estimated by the kinetic analysis in the so-called “base model” are then: *V*_c_, *k*_12_, *k*_21_, *k*_23_, *k*_32_, *k*_32_, and *k*_10_.

The hemodilution [(Hb_o_ / Hb_t_) – 1] is divided by (1– baseline hematocrit) to obtain the Hb-derived fractional plasma dilution, which corresponds to (*v*_c_ – *V*_c_) / *V*_c_. Note that the baseline Hb should be placed as numerator (Hb_o_) and the Hb measured later (Hb_t_) as denominator in this ratio to obtain the fractional plasma dilution (else the relationship between fluid volume and dilution becomes exponential). The excreted urine, whenever collected, is used as input variable for *U*. Plasma dilution based on albumin did not need correction for the hematocrit.

**Covariate models**

Individual-specific and time-specific covariates can be added to the fixed parameters and, thereby, improve the precision of the kinetic analysis in an individual participant or at a specific point in time. In the present study, three covariate models were used that all affect the fixed parameters in different ways.

The *power model* is used to analyze the influence of continuous variables on fixed parameters. In the present study, the only potential covariates for which the power model was appropriate was age, body weight, blood loss, and MAP. A prerequisite for obtaining the numerical effect of the covariance between the mean arterial pressure (MAP) and *k*_10_ we must know that the mean MAP for all experiments was 82 mmHg, the covariate parameter value was 2.10, and that the typical value (tv) of *k*_10_ was 0.0207. The value of *k*_10_ for an observation where MAP was 100 mmHg is then written:

0.0207 (100 / 82) ^2.10^ = 0.0314

which is acceleration by 50%.

The *exponential covariate model* is used to analyze the influence of categorical variables on the fixed parameters. This covariate model was used for the following potential covariates: male/female, post-infusion/ongoing infusion, Ringer´s/irrigating fluid, acetate/lactate as buffer, and awake/anesthesia. The reported typical value for the fixed parameter is the covariate that is given the value of 0, which is the first of the option in the pairs listed above. For example, if a male is denoted 0 and a female is 1, we can decide *V*_c_ for either sex by using the covariate value of -0.50 and the typical value of *V*_c_, which is 3.21. The covariate values is an exponent to the natural logarithm, e = 2.718.

Males: 3.21* (2.718 ^-0.50 * 0^ ) = 3.21, Females: 3.21* (2.718 ^- 0.50 * 1^) = 1.95

which is a reduction by almost 1/3 in the females.

The *linear covariate model* is appropriate for continuous variables that include zero or negative values. This model was used when evaluating the influence of the Hb-albumin dilution difference on a fixed parameter. If we want to calculate *k*_21_ when Hb-albumin dilution difference is +0.20, we need to know that the mean value for all paired observations was +0.004, the covariate effect 3.08, and the typical value 0.1188.

0.1188 (1 + 3.08 (0.20 – 0.004) = 0.191

which is acceleration by 60%.

The value of *k*_21_ is reduced during general anesthesia, having a covariate value of -0.50 when express according to the exponential covariate model. During general anesthesia, *k*_21_ during general anesthesia, and with a Hb-albumin dilution difference of +0.20, then becomes:

0.1188 (1 + 3.08 (0.20 – 0.004) * (2.718 ^-0.50^) = 0.1156

The criterion for accepting a covariate was that its inclusion should reduce the -2 LL (log likelihood) for the model by > 6.6 points (*P*< 0.01) and be significant both by forward addition and backward elimination. Moreover, the 95% confidence interval (CI) for the estimate of the covariate was not allowed to include 0.

_________________________________________________________________________

**
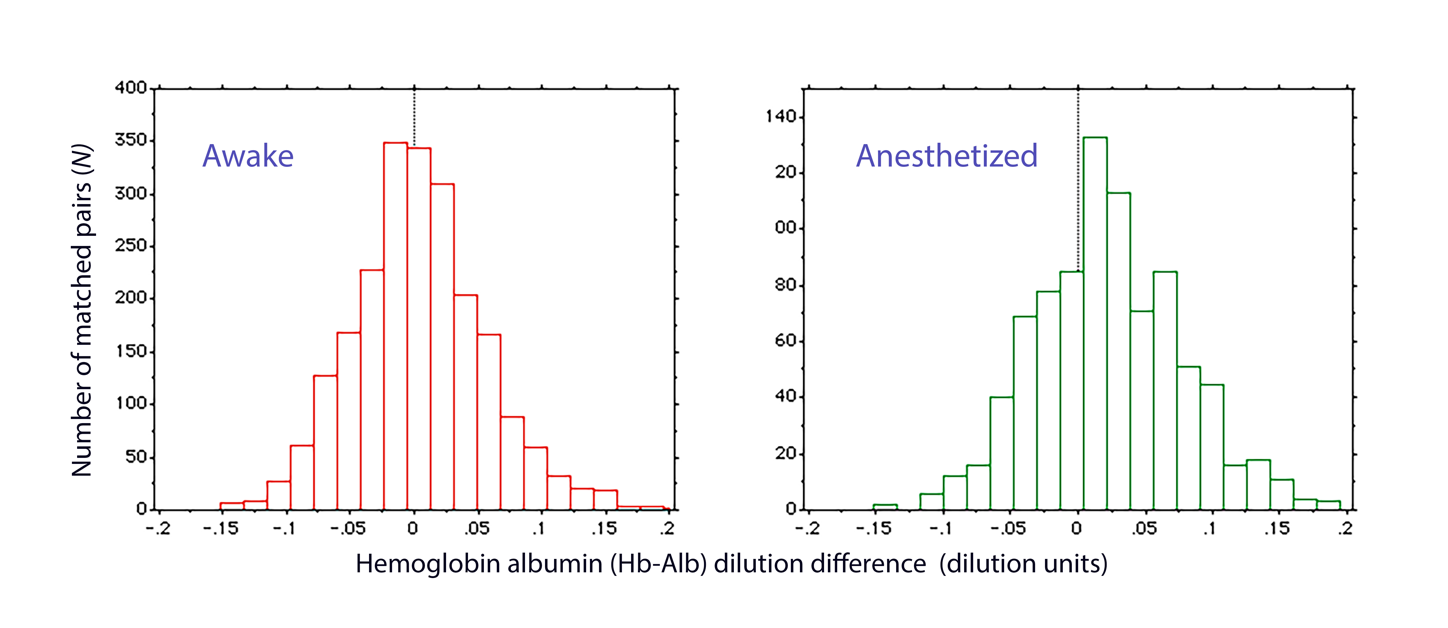
**

**Fig. S1** Distribution of the Hb-Alb dilution difference in the awake participants (left panel) and anesthetized patients (right panel). The entire experiments are shown.

**Table S1**

**Kinetic parameter values.** Same as Table 1 in the manuscript except that the development of the -2 log likelihood values can be followed for the addition of each covariate to the model.

| Kinetic parameter | Covariate | Best estimate | 95% CI | CV% | -2 LL |
| --- | --- | --- | --- | --- | --- |
| ***Fixed*** |  |  |  |  |  |
| ­­tv*V*_c_ (L) |  | 3.21 | 2.90–3.54 | 5.4 |  |
| tv*k*_12_ (10^-3^ min^-1^) |  | 113.0 | 77.1–148.0 | 16.1 |  |
| tv*k*_21_ (10^-3^ min^-1^) |  | 118.8 | 58.3–179.4 | 26.0 |  |
| tv*k*_23_ (10^-3^ min^-1^) |  | 24.0 | 20.1–28.0 | 8.4 |  |
| tv*k*_32_ (10^-3^ min^-1^) |  | 64.0 | 37.8–90.3 | 20.9 |  |
| tv*k*_10_ (10^-3^ min^-1^) |  | 20.7 | 17.5-24.1 | 8.1 | -3862 |
| ***Covariates*** |  |  |  |  |  |
| *k*_10_ | MAP (mmHg) | 2.10 | 1.52–2.68 | 14.1 | -3917 |
| *V*_c_ | Body weight (kg) | 0.75 | 0.56–0.95 | 13.4 | -3952 |
| *k*_10_ | General anesthesia (no/yes) | -1.67 | -1.98 to -1.37 | -9.4 | -4056 |
| *k*_21_ | Hb-Alb dilution difference | 3.08 | 2.00–4.15 | 17.8 | -4072 |
| *k*_12_ | Hb-Alb dilution difference | 2.86 | 1.85–3.69 | 18.0 | -4123 |
| *V*_c_ | Hb-Alb dilution difference | -1.06 | -1.56 to -0.55 | -24.4 | -4360 |
| *k*_21_ | Ongoing infusion (no/yes) | -0.50 | -0.70 to -0.30 | -20.7 | -4441 |
| *k*_23_ | Irrigating fluid (no/yes) | 1.22 | 0.85–1.60 | 15.6 | -4494 |
| *k*_23_ | Ongoing infusion (no/yes) | -7.60 | -8.49 to -6.70 | -6.0 | -4512 |
| *V*_c_ | Sex (male/female) | -0.50 | -0.62 to -0.38 | -12.1 | -4572 |
| *k*_21_ | General anesthesia (no/yes) | -0.44 | -0.61 to -0.29 | -18.2 | -4594 |

CI = confidence interval. CV% = coefficient of variation (inter-individual).

LL = log likelihood for the model. Decrease of -2 LL by >6.6 points = *P*< 0.01.

*Covariate models:* Power = MAP and body weight; Linear = Hb-Alb dilution difference; Exponential = the others.
